# Vapor transport deposition of antimony selenide thin film solar cells with 7.6% efficiency

**DOI:** 10.1038/s41467-018-04634-6

**Published:** 2018-06-05

**Authors:** Xixing Wen, Chao Chen, Shuaicheng Lu, Kanghua Li, Rokas Kondrotas, Yang Zhao, Wenhao Chen, Liang Gao, Chong Wang, Jun Zhang, Guangda Niu, Jiang Tang

**Affiliations:** 1 Sargent Joint Research Center, Wuhan National Laboratory for Optoelectronics and School of Optical and Electronic Information, Huazhong University of Science and Technology, 1037 Luoyu Road, Wuhan, 430074 Hubei China; 2 Shenzhen R&D Center of Huazhong University of Science and Technology, Shenzhen, 518000 China

## Abstract

Antimony selenide is an emerging promising thin film photovoltaic material thanks to its binary composition, suitable bandgap, high absorption coefficient, inert grain boundaries and earth-abundant constituents. However, current devices produced from rapid thermal evaporation strategy suffer from low-quality film and unsatisfactory performance. Herein, we develop a vapor transport deposition technique to fabricate antimony selenide films, a technique that enables continuous and low-cost manufacturing of cadmium telluride solar cells. We improve the crystallinity of antimony selenide films and then successfully produce superstrate cadmium sulfide/antimony selenide solar cells with a certified power conversion efficiency of 7.6%, a net 2% improvement over previous 5.6% record of the same device configuration. We analyze the deep defects in antimony selenide solar cells, and find that the density of the dominant deep defects is reduced by one order of magnitude using vapor transport deposition process.

## Abstract (web summary)

Antimony selenide possess several advantages for solar cell applications but state-of-the-art vapor transport deposition methods suffer from poor film quality. Here Wen et al. develop a fast and cheap method to reduce the defect density by 10 times and achieve a certified power conversion efficiency of 7.6%.

## Introduction

Antimony selenide (Sb_2_Se_3_) has recently emerged as a promising green alternative to CdTe solar cells because it possesses very attractive optoelectronic properties such as proper bandgap (about 1.03 eV indirect and 1.17 eV direct) for the absorption of a significant portion of the solar spectrum, high optical absorption coefficient (greater than 10^5^ cm^−1^) and decent carrier mobility (about 10 cm^2^ V^−1^ s^−1^)^1–3^. Besides, because it is a binary compound with high vapor pressure, a fast, low temperature vacuum-based deposition technique can be employed like the ones established in CdTe photovoltaics. It also possesses a one-dimensional crystal structure with loose van der Waals interaction between ribbons^1^, thereby enabling grain boundaries free of dangling bonds in *c*-axis-oriented films and minimizing recombination losses therein. Furthermore, the earth-abundant elemental compositions of Sb_2_Se_3_ as well as its easy fabrication promise low-cost manufacturing. All these attributes suggest the great potential of Sb_2_Se_3_ for high-efficiency thin film solar cell and commercial application.

However, the highest power conversion efficiency (PCE) of Sb_2_Se_3_ thin film solar cells with a CdS/Sb_2_Se_3_ superstrate configuration is so far 5.6%^2, 4–11^, and with a ZnO/Sb_2_Se_3_ superstrate configuration 5.93%^1^. Employing PbS colloidal quantum dots (QDs) as the hole transport layer, as-fabricated device CdS/Sb_2_Se_3_/PbS QDs demonstrated an efficiency of 6.5%^12^. Rapid thermal evaporation (RTE) was used to fabricate all above-mentioned Sb_2_Se_3_ solar cells. RTE is a process similar to close-space sublimation^2^, in which the vapor is limited to a confined space and rapidly deposits on the substrate. The distance from the substrate to the evaporation source is only 0.8 cm, and the whole deposition is performed within 35 s. The small confined space and the rapid deposition increase the difficulty of mixing the vapor particles (Se, Sb and Sb_*x*_Se_*y*_) evenly, potentially promoting defect formation. The formation of defects, such as interstitial and antisite defects, accelerates nonradiative recombination and degrades device performance^13–16^. Furthermore, limited by our RTE facility, a simple one-zone tube furnace, the source and substrate temperatures are strongly correlated, which seriously hinders the independent optimization of both substrate and source temperature. These problems in our RTE technique have seriously restricted the development of Sb_2_Se_3_ solar cells. Therefore, we urgently need to explore alternative strategies to further improve Sb_2_Se_3_ film quality and device performance.

Here, we develop a vapor transport deposition (VTD) process to fabricate record efficiency Sb_2_Se_3_ thin film solar cells. In the VTD process, both the substrate temperature and the distance between source and substrate are adjustable, enabling not only highly oriented Sb_2_Se_3_ film, but also enormously improved film crystallinity and reduced bulk and interfacial defects in Sb_2_Se_3_ solar cells. In addition, the VTD process is a proven low-cost and fast-turnaround manufacturing method for commercial CdTe solar cells^17, 18^, and adopting this technique for Sb_2_Se_3_ technology could facilitate the commercial competitiveness of Sb_2_Se_3_ solar cells. After optimizing the Sb_2_Se_3_ films via VTD process, we obtain the champion indium tin oxide (ITO)/CdS/Sb_2_Se_3_/Au solar cells with a certified efficiency of 7.6%, which is a record of all Sb_2_Se_3_ thin film solar cells by far^1, 2, 4–12, 19, 20^ and represents a net 2% efficiency gain over previous report with the same device configuration^2^. We comparatively analyze the physical properties of VTD-fabricated and RTE-fabricated Sb_2_Se_3_ devices by X-ray diffraction (XRD), transient absorption (TA) spectroscopy and deep-level transient spectroscopy (DLTS). We find that the VTD-fabricated devices possess much higher film crystallinity, longer carrier lifetime and fewer bulk and interfacial defects than RTE-fabricated devices. Especially, the reduced density of defect complex (Sb_Se_+Se_Sb_) enhances the device performance by decreasing the photo-generated carrier recombination.

## Results

### Fabrication of Sb_2_Se_3_ films via VTD process

**Fig. 1:**
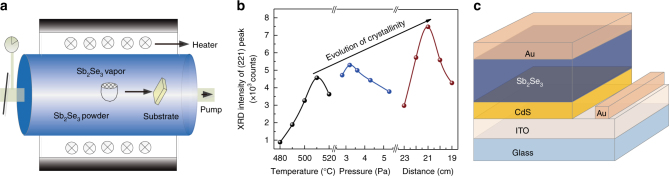
Fabrication of CdS/Sb_2_Se_3_ solar cells. **a** Schematics of our VTD system. **b** Evolution of crystallinity with the optimization of deposition condition. **c** The device structure of our Sb_2_Se_3_ solar cells

A schematic representation of VTD system is shown in Fig. 1a. The Sb_2_Se_3_ powder and the substrate were placed in the center and at the right end of the heater, respectively. The full deposition details are provided in experimental section. Hereby, the heating temperature of the evaporation source, the pressure in quartz tube and the substrate temperature are the key factors that determining the quality of final Sb_2_Se_3_ films. The distance between the substrate and the heating center was varied to achieve adjustable substrate temperature. We deposited Sb_2_Se_3_ films at different heating temperatures, pressures and substrate temperatures. XRDs of all Sb_2_Se_3_ films were measured to characterize the crystallinity and orientation of these films, which is shown in Supplementary Fig. 1. All the Sb_2_Se_3_ films had a preferred [221] orientation with the only difference being the intensity of the diffraction peaks. As Sb_2_Se_3_ is composed of one-dimensional (Sb_4_Se_6_)_*n*_ ribbons stacking together via weak van der Waals force, a proper film orientation is crucial for facile carrier transport in the film^1, 2^. Our previous reports have demonstrated that [221]-orientation enhances carrier transport across Sb_2_Se_3_ film^1, 2, 6, 8^. Therefore, with the film thickness and measurement details kept strictly identical, we utilized the intensity of (221) XRD peaks in Sb_2_Se_3_ films to evaluate their crystallinity. The crystallinity evolution of Sb_2_Se_3_ films deposited on CdS layers with varied evaporation temperature, pressure and distance was investigated, as shown in Fig. 1b. By carefully optimizing the three key factors, Sb_2_Se_3_ film with the highest crystallinity was obtained when the evaporating temperature was set at 510 °C, the pressure was 3.2 Pa and the distance from the substrate to the heating center was 21 cm. Here, the actual temperature curves of powder and substrate were monitored during the heating and cooling steps. The input temperature program and the measured temperature curves of substrate and powder are shown in Supplementary Fig. 2. This heating profile and substrate distance corresponded to the maximum actual source and substrate temperatures of 540 °C and 390 °C, respectively, as monitored by the thermocouples.

The crystallinity evolution of Sb_2_Se_3_ films indicates the importance of evaporation temperature, pressure and substrate temperature in VTD process. High evaporation temperature increases the kinetic energy of vapor particles and the surface mobility of adatoms on the substrate^21^. On the other hand, high pressure can increase in-flight collisions with background gas atoms, and then reduced the kinetic energy of vapor particles^21, 22^. In addition, increasing the substrate temperature will lead to the increased surface mobility of adatoms^22^. Hence, the kinetic energy of vapor particles and surface mobility of adatoms are ultimately defined by combination of evaporation temperature, pressure and substrate temperature. During the Sb_2_Se_3_ film deposition, vapor particles with proper kinetic energy and adatoms with proper surface mobility are mandatory for high-quality Sb_2_Se_3_ films. Energetic vapor particles and high mobility of adatoms may increase the instability of adatoms, causing displacements of lattice and consequently creating lattice defects^21, 22^. Such defects may act as new nucleation sites and increase nucleation density of adatoms^22^. In the extreme, the increased lattice defects and nucleation density may result in decreased crystallinity and quality of the film. That is why the crystallinity of the Sb_2_Se_3_ film decreased when deposited at overly high evaporation temperature, lower pressure and closer distance to evaporation source.

### Device performance

**Fig. 2:**
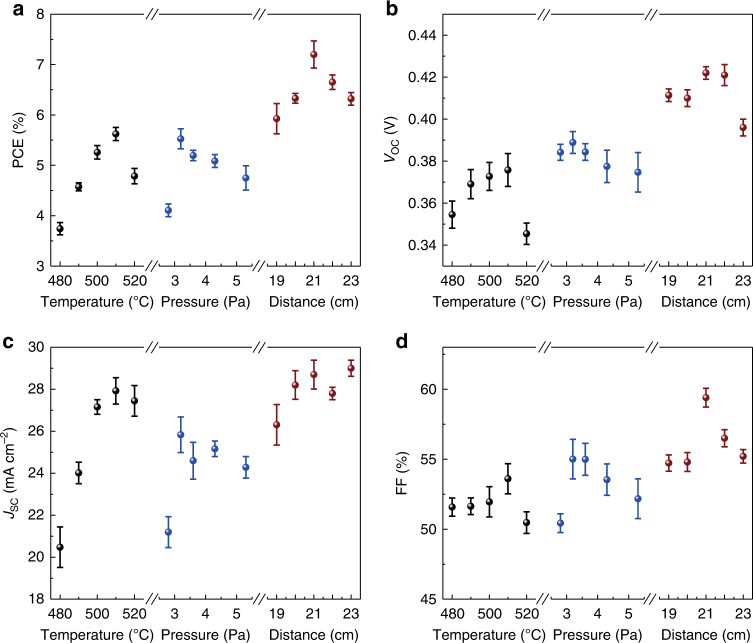
Deposition condition-dependent photovoltaic characteristics. **a** Power conversion efficiency (PCE), **b** open-circuit voltage (*V*_OC_), **c** short-circuit current density (*J*_SC_) and **d** fill factor (FF) of the CdS/Sb_2_Se_3_ solar cells fabricated by VTD process. A total of 135 devices are included for the statistics analysis. Solid sphere symbols and error bars indicate average values and standard deviations, respectively

**Fig. 3:**
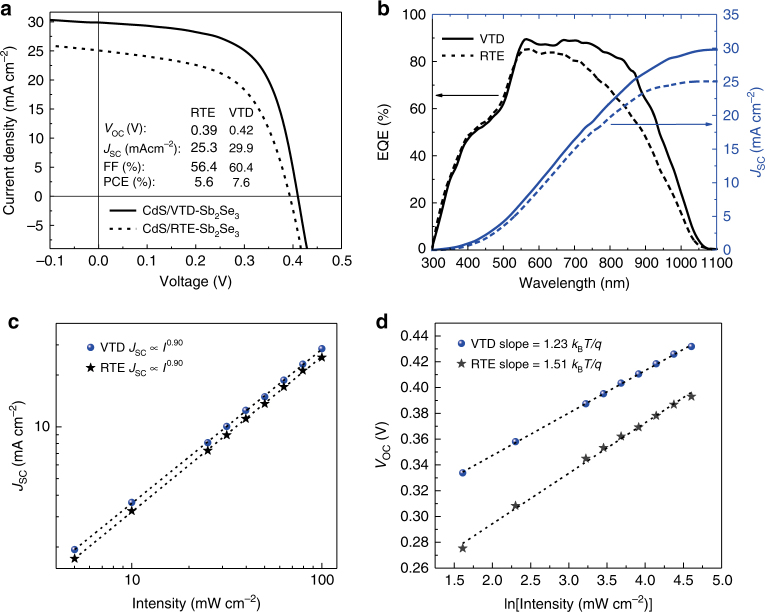
Device performance and light intensity-dependent *J*_SC_ and *V*_OC_ of devices. **a** The light *J-V* curves of VTD- and RTE-fabricated devices under AM1.5 G illumination. The *J-V* curve of the VTD-fabricated device with certified efficiency of 7.6% (area = 0.091 cm^2^) was measured by National Institute of Metrology on 1 September 2017. The calibration certificate number is GXtc2017-1987 (Supplementary Fig. 3). **b** EQE and integrated *J*_SC_ of the VTD- and RTE-fabricated devices. Light intensity-dependent **c** *J*_SC_ and **d** *V*_OC_. Neutral-density filters (THORLABS) were used to control the light intensity

The Sb_2_Se_3_ absorber layers were fabricated under the above-mentioned conditions, and the corresponding device structure ITO/CdS/Sb_2_Se_3_/Au is shown in Fig. 1c. The dependence of device performance on different evaporation temperatures, pressures and distances is summarized in Fig. 2. The variation trend of PCE is similar to the crystallinity evolution of Sb_2_Se_3_ films (Fig. 1b), which indicates that device performance strongly correlates with the crystallinity of Sb_2_Se_3_ film. As can be seen from Fig. 2, the best PCE, open-circuit voltage (*V*_OC_), short-circuit current density (*J*_SC_) and fill factor (FF) were obtained under the deposition condition where the highest film crystallinity was achieved (Fig. 1b). After carefully optimizing Sb_2_Se_3_ films and devices, the champion device with a certified power conversion efficiency of 7.6% was obtained (certificate included in Supplementary Fig. 3). This value represents the highest PCE of all Sb_2_Se_3_ thin film solar cells reported so far^1, 2, 4–12^, which is 2% higher than previous 5.6% certified efficiency with the same device configuration^2^. Figure 3a shows the light current density-voltage (*J-V*) curve of the champion VTD-fabricated CdS/Sb_2_Se_3_ solar cell with certified PCE of 7.6%, *V*_OC_ of 0.42 V, *J*_SC_ of 29.9 mA cm^−2^ and FF of 60.4%. The *J-V* curve of a RTE-fabricated solar cell with PCE of 5.6% (*V*_OC_ = 0.39 V, *J*_SC_ = 25.3 mA cm^−2^ and FF = 56.4%) is also included in Fig. 3a for comparison. Obviously, every performance parameter of the VTD-fabricated solar cell, especially *J*_SC_, is larger than that of the RTE-fabricated device.

We further checked the external quantum efficiency (EQE) of the two devices (Fig. 3b) to investigate their photo-response. The EQE spectrum of VTD-fabricated device demonstrated a higher photo-response from 520 nm (the absorption onset of CdS layer) to 1100 nm than that of RTE-fabricated device. The maximum EQE of VTD-fabricated device was close to 90% between 540 and 720 nm. For comparison, the device fabricated by our RTE technique only had the maximum EQE of about 85%. This suggests the VTD-fabricated devices possess very low recombination losses of photo-generated carriers and long carrier lifetimes at CdS/Sb_2_Se_3_ interface and in the whole Sb_2_Se_3_ absorber^23^. In Fig. 3b, we derived the current density of 29.8 and 25.1 mA cm^−2^ by integrating the EQE spectra with standard AM1.5 spectrum to further validate our *J*_SC_ values, which agreed with the experimental values of 29.9 and 25.3 mA cm^−2^ very well, respectively. Furthermore, a solar cell with initial PCE of 7.25% was stored in ambient air for about 40 days without encapsulation. This representative device was measured every week to monitor the stability of Sb_2_Se_3_ film solar cell, and the results are displayed in Supplementary Fig. 4a. The PCE remained unchanged during the whole process. For the stability of device under continuous illumination, Supplementary Fig. 4b shows slight decrease for CdS/Sb_2_Se_3_ device but no decrease for ZnO/Sb_2_Se_3_ device, which is consistent with the previous report^1^. These results indicate that VTD process is a simple and effective technique for producing high-efficiency and stable Sb_2_Se_3_ thin film solar cells.

### Carrier recombination in CdS/Sb_2_Se_3_ solar cells

From the improved EQE of VTD-fabricated devices, we inferred that the carrier transport was enhanced, and recombination loss was reduced in the active Sb_2_Se_3_ layer by VTD process. Therefore, to further clarify the carrier recombination processes in Sb_2_Se_3_ solar cells, we performed light-intensity-dependent *J*_SC_ and *V*_OC_ measurements on the VTD- and RTE-based photovoltaic devices. The complete sets of *J-V* curves are given in Supplementary Fig. 5. Figure 3c illustrates *J*_SC_ as a function of light intensity (*I*). Herein, *J*_SC_ was fitted according to the power law dependence (*J*_SC_ ∝ *I* ^*α*^) by the log–log scale plot. The power value *α* for both devices is 0.9, close to unity (first-order). This means that trap-assisted recombination is present in both solar cells and is the dominating loss mechanism^24, 25^. The light-intensity-dependent *V*_OC_ can provide critical insights into the recombination mechanism in the solar cells^26^. For *V*_OC_ measurement, the device is open circuit, so there is no current extraction from the devices, and all photo-generated carriers recombine in Sb_2_Se_3_ film. Thus, the carrier recombination process can be reflected based on the relationship of *V*_OC_ ∝ *n*(*k*_B_*T/q*)ln(*I*), where *k*_B_ is the Boltzmann constant, *T* is the temperature and *q* is elementary charge^26–29^. *n*(*k*_B_*T/q*) is the slope of *V*_OC_ vs. the natural logarithm of light-intensity ln(*I*). For trap-free solar cells, the slope of *V*_OC_ vs. ln(*I*) should be *k*_B_*T/q* (i.e., *n* = 1)^26, 29^. For our devices, as shown in Fig. 3d, the slopes obtained by linear fitting were 1.23(*k*_B_*T/q*) for the VTD-fabricated device and 1.51(*k*_B_*T/q*) for the RTE-fabricated device. This, again, indicates the presence of trap-assisted Shockley–Read–Hall (SRH) recombination in both devices^29, 30^. The slope was decreased from 1.51(*k*_B_*T/q*) to 1.23(*k*_B_*T/q*) by using VTD process, suggesting reduced trap-assisted recombination in VTD-fabricated devices^28, 31^. We measured the TA decay of the two devices to investigate the carrier lifetime (Supplementary Fig. 6). For the steady-state absorption (Supplementary Fig. 7) of VTD- and RTE-fabricated Sb_2_Se_3_ films, the absorption rose to the maximum at around 940 nm. Thus, the transient kinetic decay was monitored at 940 nm. By fitting the kinetic decay data, the longer carrier lifetime (1339 ps) was obtained in VTD-fabricated device than that (1149 ps) of RTE-derived device. Longer carrier lifetime not only permits more efficient carrier collection and hence larger *J*_SC_ (VTD 29.9 mA cm^−2^ vs. RTE 25.3 mA cm^−2^), but also enables higher carrier concentration within the absorber layer and therefore wider quasi-Fermi level splitting and improved *V*_OC_ (VTD 0.42 V vs. RTE 0.39 V).

### Morphology and crystallization of Sb_2_Se_3_ films

**Fig. 4:**
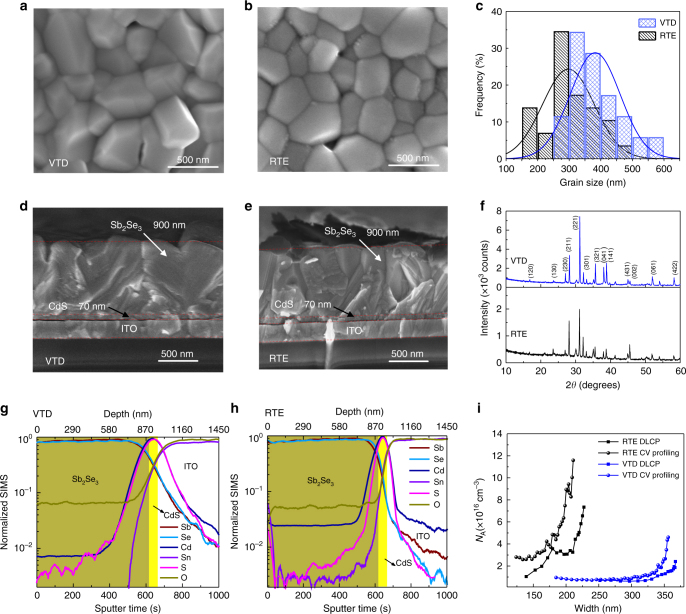
Characterization of Sb_2_Se_3_ films and interface analysis of CdS/Sb_2_Se_3_ devices. SEM top-view images of **a** VTD-fabricated and **b** RTE-fabricated Sb_2_Se_3_ films. **c** Histogram of grain size in VTD-fabricated and RTE-fabricated Sb_2_Se_3_ films. Cross-sectional SEM images of **d** VTD-fabricated and **e** RTE-fabricated CdS/Sb_2_Se_3_ devices. **f** XRD of VTD-fabricated and RTE-fabricated Sb_2_Se_3_ films. SIMS depth analysis of **g** VTD-fabricated and **h** RTE-fabricated devices. **i** *C-V* profiling and DLCP for VTD- and RTE-fabricated devices

We now study the material origin of the improved device performance. The scanning electron microscopy (SEM) top view images of Sb_2_Se_3_ films deposited on glass/ITO/CdS substrates by VTD and RTE are shown in Fig. 4a, b, respectively. The grains of VTD-fabricated film are obviously larger than that of Sb_2_Se_3_ film from RTE process. We statistically analyzed the distribution of grain size from the two SEM top view images. Figure 4c depicts the histograms of the grain size. The average grain size of VTD-fabricated film was 382 nm with a standard deviation of 79 nm, whereas the RTE-derived Sb_2_Se_3_ film had an average size of 297 nm and a standard deviation of 82 nm. The cross-sectional SEM images of Sb_2_Se_3_ solar cells fabricated by VTD and RTE methods are shown in Fig. 4d, e, respectively. Device structures in both cases were the same: ITO/CdS/Sb_2_Se_3_/Au. The Sb_2_Se_3_ layers were compact and well adherent to the CdS with the same thickness of about 900 nm. We compared XRD patterns of the two Sb_2_Se_3_ layers, measured under identical conditions, as shown in Fig. 4f. Obviously, both films were favorably orientated along [221] direction. However, the XRD intensity of VTD-fabricated film was much stronger than that of RTE-fabricated film, indicating much higher crystallinity was obtained by VTD process. The SEM and XRD results both demonstrate VTD-fabricated Sb_2_Se_3_ films have larger grains and higher crystallinity compared with RTE-fabricated films. It should be noted that, for RTE-fabricated Sb_2_Se_3_ film, the distance between evaporation source and substrate was merely 0.8 cm, and the deposition was carried out on 350 °C substrate for 35 s^1, 2^, while the corresponding value in the VTD process was 21 cm, 390 °C and 2 min, respectively. Longer traveling distance could increase the collision probability between Sb_*x*_Se_*y*_ particles and gas molecules, reduce the momentum of these particles when impinge onto the substrate and hence decrease the occurrence of nucleation. Less nucleus within the film, accompanied with higher substrate temperature and longer film growth duration, enabled the VTD-derived Sb_2_Se_3_ film with larger grain size and better grain crystallinity^21^.

### Interfacial properties of the devices

For all thin film photovoltaic devices with CdS as buffer layer, Cd diffusion at the interface is always observed^32, 33^. To check the Cd diffusion in our devices, we measured their composition distribution by secondary ion mass spectroscopy (SIMS) depth profiling. As shown in Fig. 4g, h, Cd diffusion was observed in both VTD-fabricated and RTE-fabricated CdS/Sb_2_Se_3_ devices. The deeper Cd diffusion (greater than 200 nm) than that (greater than 100 nm) in RTE-fabricated device was found in VTD-fabricated device. As CdS buffer layer was prepared following identical procedures, we deduced that the different Cd-diffused depth was certainly caused by the different deposition techniques: for VTD process, substrate temperature was about 390 °C and deposition lasted for 2 min, while in RTE process the corresponding values were 350 °C and 35 s, respectively^1, 2^. Because diffusion is driven thermally, higher substrate temperature and longer deposition time resulted in more Cd diffusion in VTD-fabricated device. As we reported before^32^, Cd diffusion converted p-type Sb_2_Se_3_ into n-type, and resulted in a buried homojunction at CdS/Sb_2_Se_3_ interface. This could reduce the interface defects and recombination at the heterojunction interface^34^, being beneficial for device performance.

Therefore, we further measured interfacial defects using capacitance-voltage (*C-V*) profiling and deep-level capacitance profiling (DLCP) techniques^1, 35, 36^. Generally, the defects density obtained from *C-V* profiling (*N*_CV_) includes the response of free carriers, and bulk and interfacial defects, while the defect density obtained from DLCP measurement (*N*_DLCP_) represents the response only from the free carrier and the bulk defects^1, 36^. Thus, we can characterize the defect density at CdS/Sb_2_Se_3_ interface by the difference between *N*_CV_ and *N*_DLCP_. As shown in Fig. 4i, obviously, the difference between *N*_CV_ and *N*_DLCP_ of RTE-fabricated device is much higher than that of VTD-fabricated device, indicating lower defect density at VTD-fabricated CdS/Sb_2_Se_3_ interface. Because the doping concentration of CdS was much higher than in Sb_2_Se_3_ film^3, 6^, almost all depletion width (*W*_d_) extended in Sb_2_Se_3_ layer. Here the volume to surface ratio is *W*_d_, the interfacial defect density of RTE-fabricated and VTD-fabricated devices was calculated to be about 2.1 × 10^11^ cm^−2^ and 2.8 × 10^10^ cm^−2^, respectively. The interfacial defect density in VTD-fabricated Sb_2_Se_3_ solar cell is also much lower than that (1.22 × 10^11^ cm^−2^) in RTE-fabricated Sb_2_Se_3_ solar cell with ZnO as buffer layer^1^. Consequently, we concluded that the promoted interfacial diffusion had effectively reduced interface defects. This is reminiscent of our previous observation that the performance of RTE-fabricated and thermally evaporated CdS/Sb_2_Se_3_ solar cells always improved when stored in ambient air for a few days^2, 6^. Similar effect has also been demonstrated in CdS/CdTe solar cells that the interfacial diffusion reduced interfacial lattice mismatch and defects, then reduced the current loss and improved device efficiency^33^.

### Deep defects in VTD- and RTE-fabricated devices

We subsequently investigated the deep defects in CdS/Sb_2_Se_3_ solar cells fabricated via VTD and RTE processes to demonstrate how the trap-assisted recombination was reduced in VTD-fabricated device. DLTS is a well-accepted powerful tool to investigate defect energy level, type and concentration in thin film photovoltaics^14, 15, 37–40^. It uses the transient capacitance of p–n junction at different temperature as a probe to monitor the changes in charge state of a deep defect center^41^. Traps in the device are filled by carriers through applying a voltage pulse to the device, which changes the capacitance associated with p–n junction of the device^42^.

**Fig. 5:**
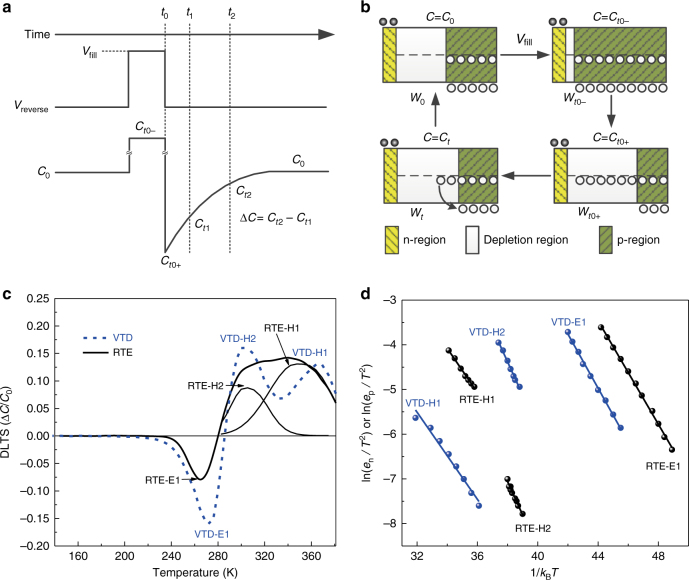
DLTS analysis of VTD- and RTE-fabricated CdS/Sb_2_Se_3_ solar cells. **a** Schematic demonstration of the mechanism of DLTS measurement. **b** Variation of depletion width and the process of holes being trapped and emitted during the measurement. **c** DLTS signals of VTD-fabricated and RTE-fabricated devices at *t*_1_/*t*_2_ = 1 ms/10 ms. **d** Arrhenius plots obtained from DLTS signals. *C*_*t*0–_ and *W*_*t*0–_ are the junction capacitance and the depletion width at the moment before pulse voltage ended, respectively. *C*_*t*0+_ and *W*_*t*0+_ are the junction capacitance and the depletion width at the moment after pulse voltage ended, respectively

We comparatively analyzed the deep-level defects in VTD-fabricated and RTE-fabricated Sb_2_Se_3_ films by DLTS. Herein, we adopted DLTS with minority carriers injection (inj-DLTS) to detect both electron and hole traps in Sb_2_Se_3_ films^37, 38, 40, 43^. As shown in Fig. 5a, during the measurement, a quiescent reverse bias (*V*_reverse_) was first applied to the junction for forming a depletion width in Sb_2_Se_3_ layer. Then, a forward pulse voltage (*V*_fill_) was applied to fill the traps in the depletion region. If we set *V*_fill_ < 0, the junction was under reverse bias, and there were only holes injected into the depletion region to fill the traps, and only hole traps were detected. If we set *V*_fill_ > 0, under forward bias condition, both electrons and holes were injected into the depletion region, and thus both electron and hole traps could be detected (inj-DLTS)^37, 38, 40^.

Here, we take the hole traps as an example to elaborate the change of transient capacitance caused by holes capture and emission (Fig. 5a). Figure 5b shows the corresponding variation of depletion width (*W*_d_) and the process of holes being trapped and emitted, before and after the pulse voltage applied. *C*_0_ represents the steady-state junction capacitance at *V*_reverse_ bias. When *V*_fill_ was applied and held for a while, *W*_d_ narrowed down to *W*_*t*0–_ and the hole trap defects were filled. Once *V*_fill_ pulse relaxed, *W*_d_ broadened to *W*_*t*0+_ and capacitance decreased to *C*_*t*0+_ instantaneously. *W*_*t*0+_ was even larger than *W*_0_ because some holes had been trapped in depletion region. Over the course of time, the trapped holes were gradually and eventually completely emitted from the occupied deep level. Then, *W*_d_ shrank to *W*_0_ and the capacitance returned to steady state (*C*_0_). As the state of activated defects was determined by the temperature, a sequence of transient capacitance was thus measured at different sample temperature, and capacitance changes within a time window vs. the different temperatures was sampled as DLTS signal. We took a fixed time window between *t*_1_ and *t*_2_ (Fig. 5a), and then the corresponding hole emission rate *e*_p_ can be expressed by Eq. (1):^15^

1 $${e}_{\mathrm{p}} = \frac{{{\mathrm{ln}}\left( {t_2/t_1} \right)}}{{t_2 - t_1}}.$$

As shown in Fig. 5a, the capacitance change within the time window is Δ*C=C*_*t*2_*−C*_*t*1_, which depends on the sample temperature. DLTS signal can be reflected from the variation of Δ*C*/*C*_0_ with different sample temperatures. Besides, based on the changing of capacitance during the discharging process of traps, the hole traps and electron traps can be differentiated by positive and negative Δ*C*, respectively.

DLTS results of VTD-fabricated and RTE-fabricated CdS/Sb_2_Se_3_ devices are shown in Fig. 5c. One negative and two positive peaks were found in both devices, indicating one electron trap (E1) and two hole trap defects (H1 and H2) in Sb_2_Se_3_ films. The activation energy and capture cross-section of traps can be obtained from Arrhenius plot based on Eqs. (2) and (3):^15, 38^

2 $${\mathrm{ln}}\left( {\frac{{e}_{\mathrm{p}}}{{T^2}}} \right) = {\mathrm{ln}}\left( {\sigma _{\mathrm{p}}\frac{{16\pi {k_{\mathrm{B}}}^2m_{\mathrm{p}}^ \ast }}{{h^3}}} \right) - \frac{{E_{\mathrm{T}} - E_{\mathrm{V}}}}{{k_{\mathrm{B}}T}},$$

3 $${\mathrm{ln}}\left( {\frac{{e_{\mathrm{n}}}}{{T^2}}} \right) = {\mathrm{ln}}\left( {\sigma _{\mathrm{n}}\frac{{16\pi {k_{\mathrm{B}}}^2m_{\mathrm{n}}^ \ast }}{{h^3}}} \right) - \frac{{E_{\mathrm{C}} - E_{\mathrm{T}}}}{{k_{\mathrm{B}}T}},$$

where *e*_n_ and *e*_p_ represents electron and hole emission rate, respectively, which can be obtained by Eq. (1); *σ*_p_ and *σ*_n_ are capture cross-section of hole and electron traps, respectively, *T* is the temperature, and *k*_B_*T* is the thermal energy. In addition, *m*_p_^*^ and *m*_n_^*^ respectively represent effective mass of hole and electron, and *E*_T_, *E*_C_ and *E*_V_ are the energy level of defect, conduction and valence bands, respectively. Figure 5d shows the Arrhenius plot obtained from Fig. 5c, by the varied *e* (hole or electron emission rate) corresponding to the DLTS peak positions in temperature according to Eqs. (2) and (3). Activation energy (*E*_T_ − *E*_V_ or *E*_C_ − *E*_T_) of the trap can be calculated from the slope of ln(*e*_p_*/T*^*2*^) or ln(*e*_n_*/T*^*2*^) vs. 1/*k*_B_*T* plot, and the capture cross-section could be extracted from the *y*-intercept. The trap concentration (*N*_T_) can be obtained from the Eq. (4)^15, 39^:

4 $$N_{\mathrm{T}} = \frac{{2\Delta C_{{\mathrm{max}}}}}{{C_0}}N_{\mathrm{A}},$$

here *N*_A_ is the net acceptor concentration in Sb_2_Se_3_ film, which can be obtained from *C-V* profiling (Fig. 4i); Δ*C*_max_ equals to the difference between *C*_*t*0+_ and *C*_0_ (Fig. 5a).

Based on the above fitting results and calculation, the defect parameters of VTD- and RTE-fabricated Sb_2_Se_3_ solar cells are summarized in Table 1. The properties of deep defects in Sb_2_Se_3_ film are experimentally uncovered. By comparing the activation energies of these trap defects, we find that they are similar in both devices, indicating the same origins of these defects. To investigate whether Cd diffusion affects the DLTS results, we measured the variation of depletion width with the applied bias, as shown in Supplementary Fig. 8. The depletion width of VTD- and RTE-fabricated devices decreased from 430 and 324 nm to 240 and 255 nm with the bias pulse changing from −0.5 V to 0.4 V, respectively. However, the Cd diffusion depths in VTD- and RTE-fabricated Sb_2_Se_3_ layers are about 200 nm and 100 nm from SIMS results, respectively. Thus, the Cd ions located outside the detected depletion region and had no effect on DLTS result. The defects only originate from the intrinsic Sb_2_Se_3_.

**Table 1:** Defect parameters of VTD- and RTE-fabricated Sb_2_Se_3_ solar cells

Defects	*E*_T_ (eV)	*σ* (cm^2^)	*N*_T_ (cm^−3^)
VTD-H1	*E*_V _+ 0.48 ± 0.07	1.5 × 10^−17^	1.2 × 10^15^
VTD-H2	*E*_V _+ 0.71 ± 0.02	4.9 × 10^−13^	1.1 × 10^14^
VTD-E1	*E*_C_ − 0.61 ± 0.03	4.0 × 10^−13^	2.6 × 10^14^
RTE-H1	*E*_V _+ 0.49 ± 0.03	2.2 × 10^−16^	1.2 × 10^14^
RTE-H2	*E*_V _+ 0.74 ± 0.04	7.7 × 10^−13^	2.3 × 10^15^
RTE-E1	*E*_C_ − 0.60 ± 0.02	1.6 × 10^−12^	1.7 × 10^15^

In our VTD and RTE facilities, Se vapor is always excess for its higher vapor pressure than Sb and Sb_2_Se_3_, so the Sb_2_Se_3_ films are actually slightly Se rich^1, 2^. Our previous ab initio calculation of the intrinsic defects in Sb_2_Se_3_ has demonstrated that the dominant acceptor defects are antimony vacancy (V_Sb_) and selenium antisite (Se_Sb_) defects under Se-rich condition^11^. Therefore, we tentatively attributed H1 and H2 defects to V_Sb_ and Se_Sb_ defects, respectively. As reported by Tumelero et al.^44^, the antisite defects dominated the distribution of defects in trichalcogenides due to the similar sizes of the constituent atoms. Our previous simulation also showed that Se_Sb_ and antimony antisite (Sb_Se_) are acceptor and donor defects, respectively^11^. Consequently, the E1 defect is most likely associated with the formation of Sb_Se_ antisite defects. Interestingly, E1 and H2 always have similar densities with each other in both VTD- and RTE-fabricated samples, which indicate that antisite defect pairs formed in Sb_2_Se_3_ films, presumably forming [Sb_Se_+Se_Sb_] complex. Please also note that using VTD process reduced the density of these antisite defect pairs by more than an order of magnitude (Table 1).

**Fig. 6:**
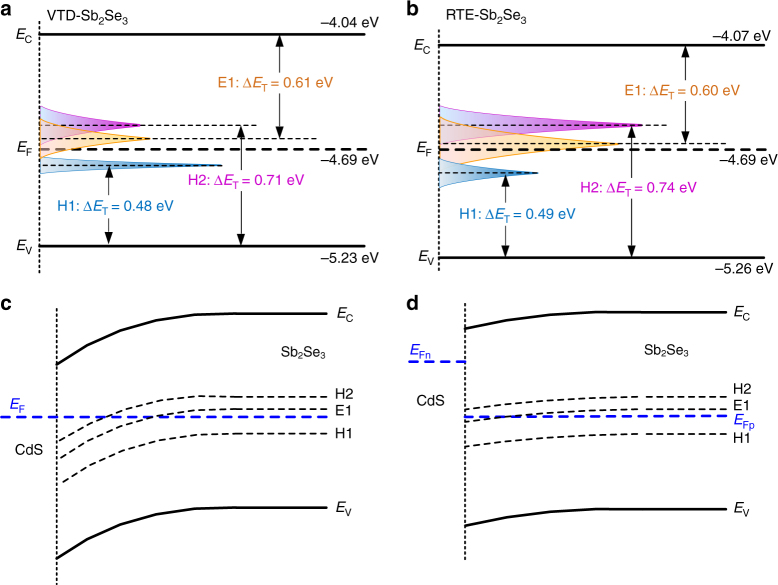
Influence of defect levels on the CdS/Sb_2_Se_3_ solar cells. Energy states and defect level of **a** VTD-fabricated and **b** RTE-fabricated Sb_2_Se_3_ films. Energy band diagrams at CdS/Sb_2_Se_3_ interface **c** in the dark and **d** under illumination

The energy levels of defects in the two samples are depicted in Fig. 6a, b, respectively. Herein, the conduction band, valence band and Fermi levels were obtained from ultraviolet photoelectron spectroscopy (UPS) and Tauc plots by transmission spectra of Sb_2_Se_3_ films (Supplementary Fig. 9). Clearly, the energy levels of H2 and E1 are above the Fermi level (*E*_F_) in both samples, and H1 is under *E*_F_. To analyze the influence of defects on the photo-generated carrier recombination, energy band and defect energy levels of CdS/Sb_2_Se_3_ device in the dark and under illumination are depicted in Fig. 6c, d, respectively. Due to thermal equilibrium, the energy band of Sb_2_Se_3_ bended downward around the hetero-interface, which led to the same Fermi level in CdS and Sb_2_Se_3_ layers. Under illumination, as depicted in Fig. 6d, the photo-generated electrons were driven into CdS layer, prompting electron quasi-Fermi level (*E*_Fn_) to move upward in CdS. Meanwhile the photo-generated holes resulted in the hole quasi-Fermi level (*E*_Fp_) shifting downward in Sb_2_Se_3_ layer. The shift of quasi-Fermi levels is positively correlated with the nonequilibrium carrier concentration. The difference between the two quasi-Fermi levels determines *V*_OC_ of the solar cell^14^.

We now discuss influence of defects in working conditions. The position of *E*_Fp_ is always dependent on illumination intensity, and Fig. 6d revealed the situation under AM1.5 irradiation. The intersections of *E*_F_ and defect levels can be used as boundaries to differentiate whether the defects are charged or not during the shifting of quasi-Fermi level^45^. Clearly, H1 state is under Fermi level and submersed in electrons, so H1 defects always stay inert. In contrast, H2 and E1 defect states are mostly above *E*_F_ and they are active in trapping holes and electrons, respectively. These trapped photo-generated carriers would most probably contribute to recombination loss. Furthermore, the energy levels of H2 and E1 are located near to the midgap, which significantly increase the recombination possibility of photo-generated carriers^46^. Therefore, the H2 and E1 are the dominant defects that influence the shift of quasi-Fermi levels and trap-assisted recombination, and then the *V*_OC_ and *J*_SC_ of the solar cells. Moreover, due to the higher defect density of H2 and E1 than carrier concentration (about 10^13^ cm^−3^) in Sb_2_Se_3_ layer^3^, the *E*_Fp_ would be more likely to be pinned near E1 and H2 levels. Obviously, both of H2 and E1 have lower defect density in VTD-fabricated sample, which could lead to relatively larger *E*_Fp_ downshifting and suppressed trap-assisted recombination, explaining the better *V*_OC_ and *J*_SC_ in VTD-fabricated Sb_2_Se_3_ solar cells.

## Discussion

We have demonstrated that VTD technique can greatly enhance the performance of Sb_2_Se_3_ thin film solar cells by increasing the crystallinity of Sb_2_Se_3_ films, reducing the interface and bulk defects in the devices, and prolonging the carrier lifetime. Specifically, we believe the advantages of VTD over RTE are based on two features. First, the substrate temperature in VTD process can be regulated independently by changing the distance between source and substrate, thus permitting higher substrate temperature (VTD 390 °C vs. RTE 350 °C). The higher substrate temperature resulted in improved crystallinity (threefold enhancement in XRD peak intensity) and average grain size (VTD 382 nm vs. RTE 297 nm) of Sb_2_Se_3_ films. Second, the slower deposition of Sb_2_Se_3_ films (VTD 2 min vs. RTE 35 s) enables less film imperfection (Se_Sb_+Se_Sb_ antisite complex and V_Sb_ vacancy), as reflected by the reduced bulk defect density (VTD 10^14^ cm^−3^ vs. RTE 10^15^ cm^−3^) and interfacial defect density (VTD 2.8 × 10^10^ cm^−2^ vs. RTE 2.1 × 10^11^ cm^−2^), and increased carrier lifetime (VTD 1339 ps vs. 1149 ps). Overall, all these advantages facilitated the fabrication of high-quality Sb_2_Se_3_ films, leading to increased *V*_OC_, *J*_SC_ as well as FF (VTD: 0.42 V, 29.9 mA cm^−2^, 60.4% vs. RTE: 0.39 V, 25.3 mA cm^−2^, 56.4%). The champion device achieved the efficiency record 7.6%, much higher than SnS, Cu_2_O and FeS_2_ solar cells which have been studied for many years^47–49^. The fabrication of record efficiency Sb_2_Se_3_ solar cells employing vapor transport deposition, a technique with proven high turnaround and low cost for commercial CdTe solar cells, further strengthens the great potential of our Sb_2_Se_3_ thin film photovoltaics.

On the other hand, we find that the deep defect density in the best-performing Sb_2_Se_3_ solar cells is about 10^14^ to 10^15^ cm^−3^, which is much higher than that (10^11^ to 10^13^ cm^−3^) in CdTe solar cells^50^. These abundant deep defects could pin the quasi-Fermi level near H2 and E1 defect states, which provides a plausible explanation for the low *V*_OC_ observed in all Sb_2_Se_3_ solar cells reported so far, despite various device configuration and film preparation methods have been explored^1–12, 19, 32, 51^. We suggest that future research on tightly controlling Se and Sb components in the vapor, their strictly stoichiometric condensation into the film and the growth of highly crystalline film should be carried out to minimize these deep defects and maximize device performance.

In summary, we have obtained the superstrate CdS/Sb_2_Se_3_ thin film solar cell with a certified efficiency of 7.6% (*V*_OC_ of 0.42 V, *J*_SC_ of 29.9 mA cm^−2^ and FF of 60.4%), which was promoted by the VTD-fabricated Sb_2_Se_3_ absorber layer. Compared with the previous reports on Sb_2_Se_3_ solar cells^1–6, 8–12, 19, 20^, VTD process reduced the density of deep defects and subsequently suppressed trap-assisted recombination in Sb_2_Se_3_ films, resulting in longer carrier lifetime and better device performance. These encouraging results highlight the potential of Sb_2_Se_3_ solar cells for high-efficiency photovoltaic devices.

## Methods

### Sb_2_Se_3_ solar cell fabrication

All solar cells were deposited on ITO (In_2_O_3_:Sn) transparent conductive glass supplied by Kaivo, with sheet resistance of 6.5 to 6.8 ohm sq^−1^, transmittance of 78.8 to 79.6% and ITO thickness of about 200 nm. The ITO substrates were cleaned using detergent, acetone, isopropanol, ethanol and deionized water in sequence. CdS buffer layer was deposited by chemical bath deposition^7^. CdS layers were treated with H_2_O_2_ (30 wt%) and 20 mg ml^−1^ CdCl_2_ (Aladdin) absolute methanol solution by spin coating, respectively, baked on the hotplate at 400 °C for 5 min in air ambient and then cooled down naturally. Following that, VTD process was used to deposit Sb_2_Se_3_ films. As shown in Fig. 1a, 0.25 g Sb_2_Se_3_ powder (99.999% purity, Jiangxi Ketai) was put into a quartz crucible and placed in the center of VTD system (a single temperature zone tube furnace, MTI, Hefei, China). The ITO/CdS substrate was immobilized on a graphite support and then placed at the right end of the quartz tube. Substrate temperature was regulated by changing the distance between substrate and the center of the heater. Vacuum was pumped by a mechanical pump and the stabilized chamber pressure was controlled by varying the ventilation power of the pump. The heating temperature of VTD system was raised to the targeted evaporation temperature with a ramp rate of 20 °C min^−1^ and kept for 2 min to obtain a desired Sb_2_Se_3_ film thickness. Then, we turned off the power to stop the deposition and finally took the sample out when it was naturally cooled down to about 100 °C. After that, gold back-contact electrodes (0.091 cm^2^ area, 100 nm thick) were evaporated by the resistance evaporation thin film system (Beijing Technol Science) under a vacuum pressure of 5 × 10^−3^ Pa. To optimize the quality of Sb_2_Se_3_ film and the performance of device, we systematically investigated the evaporation temperature of VTD system from 480 to 520 °C, the pressure from 2.8 to 5.3 Pa and the substrate distance from 19 to 23 cm. When the evaporation temperature was investigated, the pressure and the distance were set as 4 Pa and 22 cm, respectively. The optimized evaporation temperature and the distance of 22 cm were adopted for pressure investigation, and then the optimized temperature and pressures were used to investigate the substrate distance.

### Material characterization

Material and device characterization are similar to previous report^1, 2, 11^. XRD of Sb_2_Se_3_ films was performed using a Philips X’Pert Pro diffractometer with Cu Kα radiation (*λ* = 1.54 Å). SEM measurement was carried out with FEI Nova Nano SEM450. SIMS (IMS-4f, CAMECA instruments) was used to investigate the element distribution along the depth in the devices. UPS was used to investigate the Fermi level and valence band of Sb_2_Se_3_ films. Experiments were performed using a He I (21.21 eV) gas discharge lamp in a Kratos AXIS-ULTRA DLD-600W x-ray photoelectron spectroscopy measurement system and recorded at 0 V samples bias in an ultrahigh vacuum chamber. The surface of Sb_2_Se_3_ films was etched before UPS measurement. Ultraviolet–visible near-infrared transmission spectra (Perkin Elmer Instrument, Lambda 950 using integrating sphere) were measured to determine the bandgap of Sb_2_Se_3_ films.

### Device characterization

*J-V* curve and PCE of the champion CdS/Sb_2_Se_3_ solar cell was independently measured by National Institute Metrology (NIM), using the Class AAA Solar Simulator with double-light source (SAN-EI ELECTRIC, XHS-2350M1, 100 mW cm^−2^, AM1.5 illumination) in air ambient at room temperature. A metal mask was used to define the area of incident light. The area (9.099 mm^2^) was also measured by NIM. The light intensity was calibrated by a standard Si-reference solar cell. A Keithley 2400 Source Meter was used to acquire *J-V* data. The external quantum efficiency of solar cells was measured using the light source generated by a 300 W xenon lamp of Newport (Oriel, 69911) and then split into specific wavelengths by a Newport Oriel Cornerstone 130 1/8 Monochromator (Oriel, model 74004). A standard silicon detector (70356_70316NS_455) was used for the calibration. The capacitance-voltage (*C-V*) profiling and DLCP data were measured using Keithley 4200. *C-V* measurements were performed at room temperature in an electromagnetic shielding box at a frequency of 100 kHz and a.c. amplitude of 30 mV. The d.c. bias voltage was scanned from −1.0 V to 0.3 V. DLCP measurements were performed with a.c. amplitude from 0.02 V to 0.14 V and d.c. bias voltage from −0.2 V to 0.2 V. DLTS measurement were performed by Semetrol DLTS system (Semetrol, LLC, USA) on the two typical devices. The temperature was scanned between 100 and 380 K, at a heating rate of 2 K min^−1^. The reverse bias voltage was −0.5 V. The filling pulse voltage and width were 0.4 V and 1 ms, respectively. At every scanning temperature point, the transient capacitance was measured 20 times for obtaining the average value. Transient absorption spectroscopy was pumped by 500 nm laser pulses (Light Conversion, Pharos, 350 fs duration pulses, 5 kHz repetition rate) and probed at 940 nm. The time delay was adjusted by changing the path length of the probe.

### Data availability

The data that support the findings of this study are available from the corresponding author on request.

## Electronic supplementary material

Supplementary Information

## References

[1] Wang L (2017). Stable 6%-efficient Sb_2_Se_3_ solar cells with a ZnO buffer layer. Nat. Energy.

[2] Zhou Y (2015). Thin-film Sb_2_Se_3_ photovoltaics with oriented one-dimensional ribbons and benign grain boundaries. Nat. Photon..

[3] Chen C (2017). Characterization of basic physical properties of Sb_2_Se_3_ and its relevance for photovoltaics. Front. Optoelectron..

[4] Liu X (2014). Thermal evaporation and characterization of Sb_2_Se_3_ thin film for substrate Sb_2_Se_3_/CdS solar cells. ACS Appl. Mater. Interfaces.

[5] Leng M (2014). Selenization of Sb_2_Se_3_ absorber layer: an efficient step to improve device performance of CdS/Sb_2_Se_3_ solar cells. Appl. Phys. Lett..

[6] Luo M (2014). Thermal evaporation and characterization of superstrate CdS/Sb_2_Se_3_ solar cells. Appl. Phys. Lett..

[7] Wang L (2015). Ambient CdCl_2_ treatment on CdS buffer layer for improved performance of Sb_2_Se_3_ thin film photovoltaics. Appl. Phys. Lett..

[8] Liu X (2015). Improving the performance of Sb2Se3 thin film solar cells over 4% by controlled addition of oxygen during film deposition. Prog. Photo. Res. Appl..

[9] Li Z (2017). Sb_2_Se_3_ thin film solar cells in substrate configuration and the back contact selenization. Sol. Energy Mater. Sol. Cells.

[10] Yuan C, Jin X, Jiang G, Liu W, Zhu C (2016). Sb_2_Se_3_ solar cells prepared with selenized dc-sputtered metallic precursors. J. Mater. Sci. Mater. Electron.

[11] Liu X (2017). Enhanced Sb2Se3 solar cell performance through theory-guided defect control. Prog. Photo. Res. Appl..

[12] Chen C (2017). 6.5% certified Sb2Se3 solar cells using PbS colloidal quantum dot film as hole transporting layer. ACS Energy Lett..

[13] Leijtens T (2016). Carrier trapping and recombination: the role of defect physics in enhancing the open circuit voltage of metal halide perovskite solar cells. Energy Environ. Sci..

[14] Heo S (2017). Deep level trapped defect analysis in CH_3_NH_3_PbI_3_ perovskite solar cells by deep level transient spectroscopy. Energy Environ. Sci..

[15] Lang DV (1974). Deep-level transient spectroscopy: a new method to characterize traps in semiconductors. J. Appl. Phys..

[16] Yang WS (2017). Iodide management in formamidinium-lead-halide-based perovskite layers for efficient solar cells. Science.

[17] Kestner JM (2004). An experimental and modeling analysis of vapor transport deposition of cadmium telluride. Sol. Energy Mater. Sol. Cells.

[18] Wangperawong A (2014). Bifacial solar cell with SnS absorber by vapor transport deposition. Appl. Phys. Lett..

[19] Ngo TT (2014). Electrodeposition of antimony selenide thin films and application in semiconductor sensitized solar cells. ACS Appl. Mater. Interfaces.

[20] Choi YC (2014). Sb_2_Se_3_-Sensitized inorganic-organic heterojunction solar cells fabricated using a single-source precursor. Angew. Chem. Int. Ed..

[21] Mattox DM (1989). Particle bombardment effects on thin-film deposition: A review. J. Vac. Sci. Technol. A.

[22] Rossnagel SM (2003). Thin film deposition with physical vapor deposition and related technologies. J. Vac. Sci. Technol. A.

[23] Lin XZ (2016). 11.3% efficiency Cu(In,Ga)(S,Se)_2_ thin film solar cells via drop-on-demand inkjet printing. Energy Environ. Sci..

[24] Quilettes DW (2015). Impact of microstructure on local carrier lifetime in perovskite solar cells. Science.

[25] Sun ZH, Sitbon G, Pons T, Bakulin AA, Chen ZY (2015). Reduced carrier recombination in PbS-CuInS_2_ quantum dot solar cells. Sci. Rep..

[26] Zhao BD (2017). High open-circuit voltages in Tin-rich low-bandgap perovskite-based planar heterojunction photovoltaics. Adv. Mater..

[27] Gao F (2014). Trap-induced losses in hybrid photovoltaics. ACS Nano..

[28] Abbaszadeh D (2016). Elimination of charge carrier trapping in diluted semiconductors. Nat. Mater..

[29] Szendrei K, Gomulya W, Yarema M, Heiss W, Loi MA (2010). PbS nanocrystal solar cells with high efficiency and fill factor. Appl. Phys. Lett..

[30] Li YW (2016). High-efficiency robust perovskite solar cells on ultrathin flexible substrates. Nat. Commun..

[31] Jahandar M (2016). Highly efficient metal halide substituted CH_3_NH_3_I(PbI_2_)_1-x_(CuBr_2_)_x_ planar perovskite solar cells. Nano Energy.

[32] Zhou Y (2017). Buried homojunction in CdS/Sb_2_Se_3_ thin film photovoltaics generated by interfacial diffusion. Appl. Phys. Lett..

[33] Kumar SG, Rao KSRK (2014). Physics and chemistry of CdTe/CdS thin film heterojunction photovoltaic devices: fundamental and critical aspects. Energy Environ. Sci..

[34] Liu FY (2016). Nanoscale microstructure and chemistry of Cu_2_ZnSnS_4_/CdS interface in Kesterite Cu_2_ZnSnS_4_ solar cells. Adv. Energy Mater..

[35] Heath JT, Cohen JD, Shafarman WN (2004). Bulk and metastable defects in CuIn_1−x_Ga_x_Se_2_ thin films using drive-level capacitance profiling. J. Appl. Phys..

[36] Duan HS (2013). The role of sulfur in solution-processed Cu_2_ZnSn(S,Se)_4_ and its effect on defect properties. Adv. Funct. Mater..

[37] Auret FD, Nel M (1987). Detection of minority-carrier defects by deep level transient spectroscopy using Schottky barrier diodes. J. Appl. Phys..

[38] Fourches N (1991). A quantitative treatment for deep level transient spectroscopy under minority-carrier injection. J. Appl. Phys..

[39] Kerr LL (2004). Investigation of defect properties in Cu(In,Ga)Se_2_ solar cells by deep-level transient spectroscopy. Solid-State Electron.

[40] Fleming RM, Seager CH, Lang DV, Campbell JM (2015). Injection deep level transient spectroscopy: An improved method for measuring capture rates of hot carriers in semiconductors. J. Appl. Phys..

[41] Khan, A. et al. DLTS: A promising technique for the identification of the recombination and compensator centers in solar cell materials. 1763–1768 (Conference Record of the 2006 IEEE 4th World Conference on Photovoltaic Energy Conversion, Waikoloa, 2006).

[42] Nguyen TP (2008). Defects in organic electronic devices. Phys. Stat. Sol. (a).

[43] Dharmarasu N (2002). Majority- and minority-carrier deep level traps in proton-irradiated n+/p-InGaP space solar cells. Appl. Phys. Lett..

[44] Tumelero MA, Faccio R, Pasa AA (2016). Unraveling the native conduction of trichalcogenides and its ideal band alignment for new photovoltaic interfaces. J. Phys. Chem. C.

[45] Boix PP (2009). Determination of gap defect states in organic bulk heterojunction solar cells from capacitance measurements. Appl. Phys. Lett..

[46] Sherkar TS (2017). Recombination in perovskite solar cells: significance of grain boundaries, interface traps, and defect ions. ACS Energy Lett..

[47] Sinsermsuksakul P (2014). Overcoming efficiency limitations of SnS-Based solar cells. Adv. Energy Mater..

[48] Minami T, Nishi Y, Miyata T (2015). Heterojunction solar cell with 6% efficiency based on an n-type aluminum-gallium-oxide thin film and p-type sodium-doped Cu_2_O sheet. Appl. Phys. Express.

[49] Prabukanthan P, Thamaraiselvi S, Harichandranb G (2017). Single step electrochemical deposition of p-type undoped and Co^2+^ doped FeS_2_ thin films and performance in heterojunction solid solar cells. J. Electrochem. Soc..

[50] Versluys J, Clauws P, Nollet P, Degrave S, Burgelman M (2004). Characterization of deep defects in CdS/CdTe thin film solar cells using deep level transient spectroscopy. Thin Solid Films.

[51] Liang GX (2018). Thermally induced structural evolution and performance of Sb_2_Se_3_ films and nanorods prepared by an easy sputtering method. Sol. Energy Mater. Sol. Cells.

