# Supplementary material for: Vapor transport deposition of antimony selenide thin film solar cells with 7.6% efficiency
Source: Nat Commun. 2018 Jun 5;9:2179. doi: 10.1038/s41467-018-04634-6 (PMC5988661; doi:10.1038/s41467-018-04634-6)
Supplement: Supplementary file 1 — Supplementary Information [file 41467_2018_4634_MOESM1_ESM.pdf]

Vapor transport deposition of Sb_2Se_3 thin film solar cells with 7.6% efficiency

Wen et al.

Supplementary Figure 1

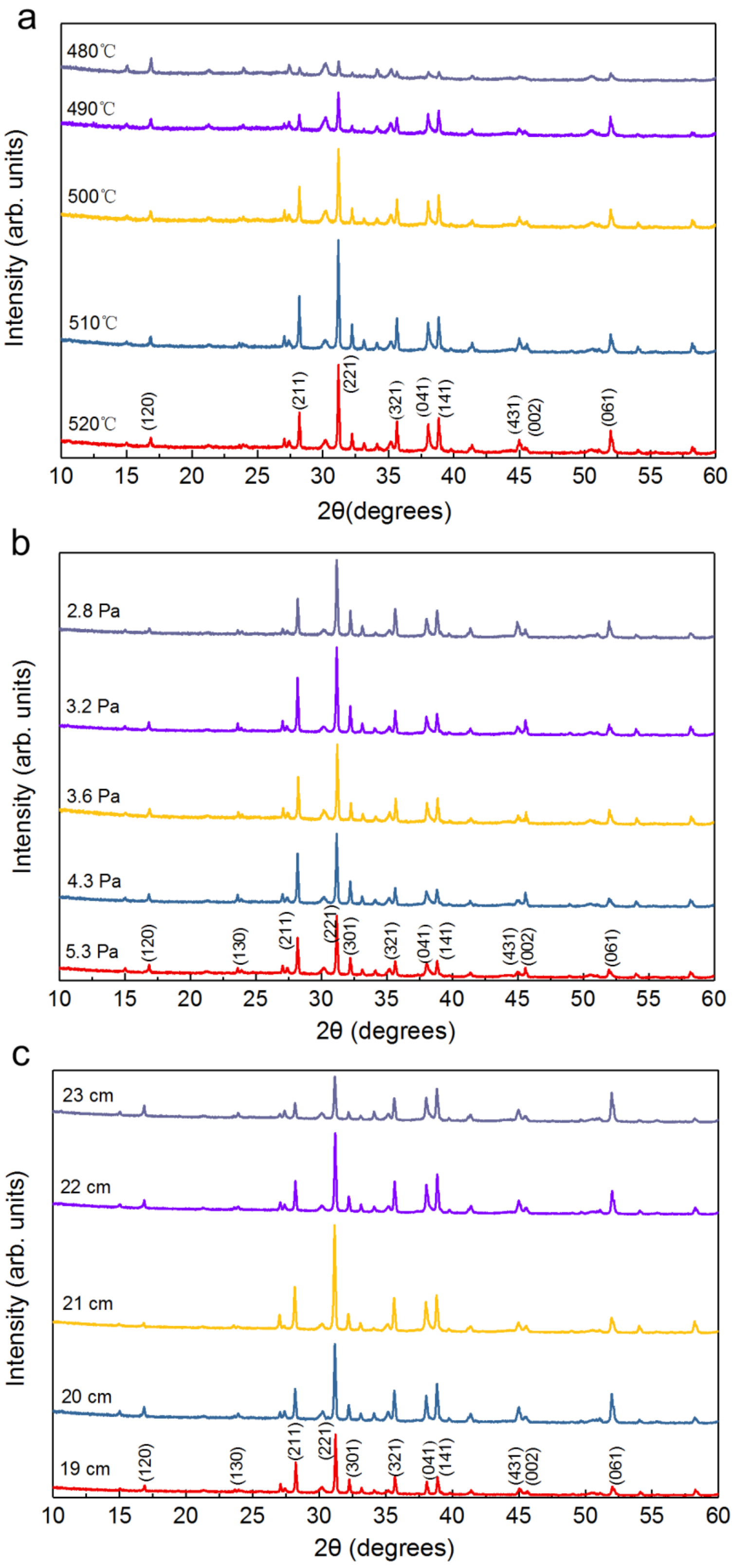

X-ray diffraction patterns of VTD fabricated Sb_2Se_3 films on CdS substrate. XRD of Sb_2Se_3 films deposited (a) at different evaporation temperature, (b) at different ambient pressure and (c) with different distance from substrate to the center of heater.

Supplementary Figure 2

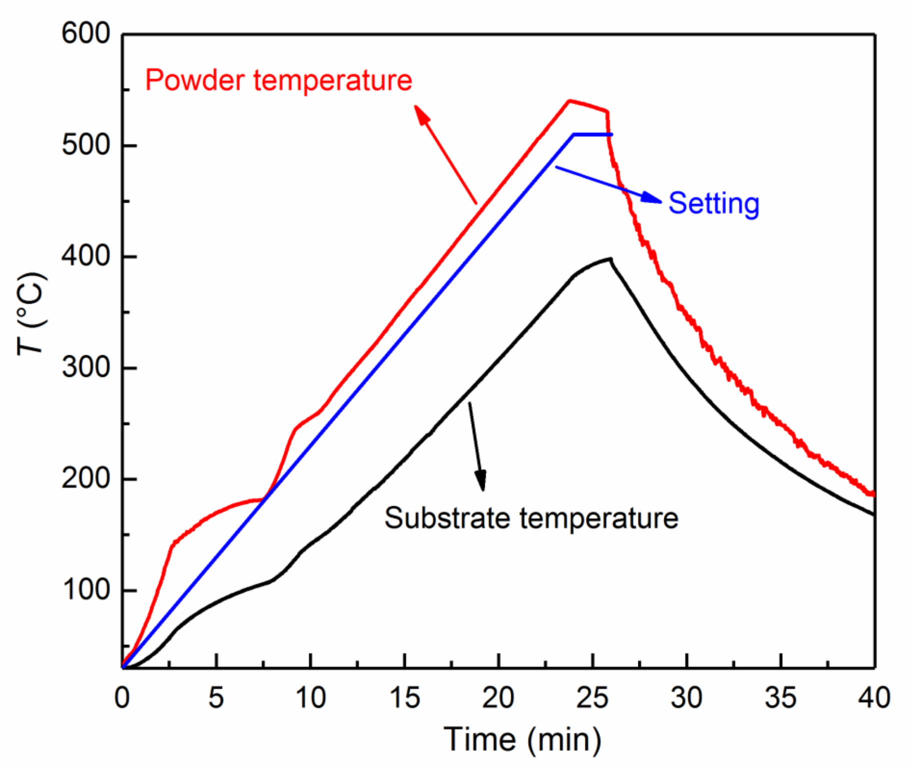

The temperature varying curves of Sb_2Se_3 powder and substrate during the optimized deposition procedure.

Supplementary Figure 3

中 国 计 量 科 学 研 究 院

测 试 报 告
Test Report

证书编号 GXtc2017-1987
Certificate No.

客户名称<br>Client	华中科技大学<br>Huazhong University of Science and Technology
器具名称<br>Instrument	硒化锑薄膜太阳能电池<br>Sb2Se3 thin film solar cell
型号/规格<br>Type/Model	ITO/CdS/Sb2Se3/Au
出厂编号<br>Serial No.	7061 -01
生产厂家<br>Manufacturer	Huazhong University of Science and Technology
客户地址<br>Address	武汉市珞瑜路 1037 号 （1037# Luoyu Road, Wuhan, 430074, Hubei, P. R. China）
测试日期<br>Date of Test	2017-09-01

批准人：
Approved by [illegible]

中国计量科学研究院 1 测试专用章

地址：中国 北京 北三环东路 18 号
Address: No.18 Bei San Huan Dong Lu,Beijing,P.R.China

邮编：100029
Post Code

电话：+86-10-64525569/74
Tel

传真：+86-10-64271948
Fax

网址：http://www.nim.ac.cn
Website

电子邮箱：kehufuwu@nim.ac.cn
Email

第 1 页 共 4 页

中 国 计 量 科 学 研 究 院

证书编号 GXtc2017-1987
Certificate No.

中国计量科学研究院是国家最高的计量科学研究中心和国家级法定计量技术机构。1999 年授权签署了国际计量委员会（CIPM）《国家计量基(标)准和国家计量院签发的校准与测量证书互认协议》(CIPM MRA)。The National Institute of Metrology (NIM) is China’s national metrology institute (NMI) and a state-level legal metrology institute. NIM is China’s signatory to the Mutual Recognition of National Measurement Standards and of Calibration and Measurement Certificates Issued by National Metrology Institutes (CIPM MRA) which is arranged by the International Committee of Weights and Measures (CIPM).<br>中国计量科学研究院的质量管理体系符合 ISO/IEC17025 标准，通过中国合格评定国家认可委员会和亚太计量规划组织（APMP）联合评审的校准和测量能力（CMCs）在国际计量局（BIPM）关键比对数据库中公布。NIM’s quality management system meets requirements of the ISO/IEC 17025. Its Calibration and Measurement Capabilities (CMCs) that are peer reviewed both by China National Accreditation Service for Conformity Assessment (CNAS) and the Asia Pacific Metrology Programme (APMP) are published in the International Bureau of Weights and Measures (BIPM) Key Comparison Database (KCDB).<br>2011 年，中国计量科学研究院和中国合格评定国家认可委员会就认可领域的技术评价活动签署了谅解备忘录，承认中国计量科学研究院的计量支撑作用和出具的校准/检测结果的溯源效力。NIM and CNAS signed a Memorandum of Understanding (MOU) for Recognition of Technical Assessment in Laboratory Accreditation Field in 2011, in which CNAS recognizing the technical supporting role of NIM in laboratory accreditation and the traceability of NIM's calibration / test results.<br>测试结果不确定度的评估和表述均符合 JJF1059 系列标准的要求。The evaluation and expression of uncertainty of the test results are in line with the requirements of JJF1059 series standards.
测试所依据的技术文件（代号、名称）Reference documents (Code,Name)<br>Measurement of photovoltaic current-voltage characteristics（IEC60904-1）JJF 加上去<br>太阳电池测试规范：光电性能（NIM-ZY-GX-TT-402）(Test Specification of Solar Cells: Photoelectric Properties)<br>太阳电池校准规范：光电性能（JJF 1622-2017）(Calibration Specification of Solar Cells: Photoelectric Properties)
测试环境条件及地点 Test place and environment<br>温 度 Temperature：25 ℃ 地 点 Location： 光学楼 110 室<br>湿 度 Humidity：45 %RH 其 它 Others：
测试使用的计量基（标）准装置(含标准物质)/主要仪器<br>Reference Standards（Including the Reference Material）/ Instruments used

名 称<br>Name	测量范围<br>Measurement Range	不确定度/<br>准确度等级<br>Uncertainty/Accuracy	证书编号<br>Certificate No.	证书有效期至<br>Due Date<br>(YYYY-MM-DD)
太阳电池光电性能校准装置<br>Measurement Standard	I_{sc}:（0.1-10）A<br>V_{oc}: (0.1-200)V<br>P_m:(0.01-500) W	I_{sc}: 1.5%（k=2）<br>V_{oc}: 0.5% (k=2)<br>P_m:1.6% (k=2)	[2015]国量标计证字第 286 号	2019-07-05
标准太阳电池<br>Reference solar cell	I_{sc}:（0-200）mA	1.2% (k=2)	GXtc2017-0782	2018-03-16

第 2 页 共 4 页

证书编号 GXtc2017-1987
Certificate No.

测试结果
Calibration Results

1. 测试条件 Test Conditions:

标准太阳电池：单晶硅（81#）；
Reference Solar Cell: mono-Si（81#）；
标准太阳电池的标定值：125.68 mA；
CV of Reference Solar Cell：125.68 mA；
太阳模拟器：双光源太阳模拟器，AAA 级；
Solar Simulator Classification：double-light source in AAA classification;
温度传感器/控制系统：无；
Teperature Sensor/Control System: None;
扫描方向：正扫
Scan Direction: Forward;
Mask:9.099mm^2

2. I-V 特性参数 I-V Characteristic parameters:

以上述标准太阳电池标定太阳模拟器辐照度至 1000 W/m^2，校准被测太阳电池的 I-V 特性曲线和参数如下：

By using the above reference solar cell to calibrate the solar simulator's irradiance to 1000 W/m^2. Measured I-V characteristic curve and parameters as follows:

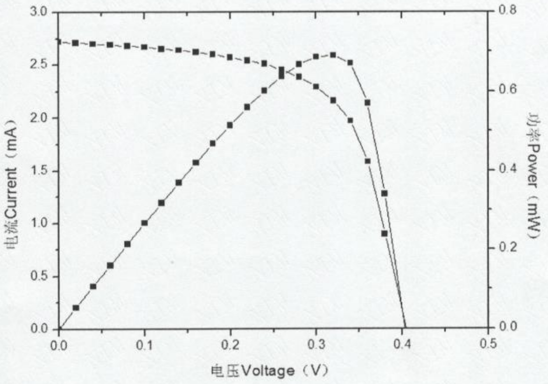

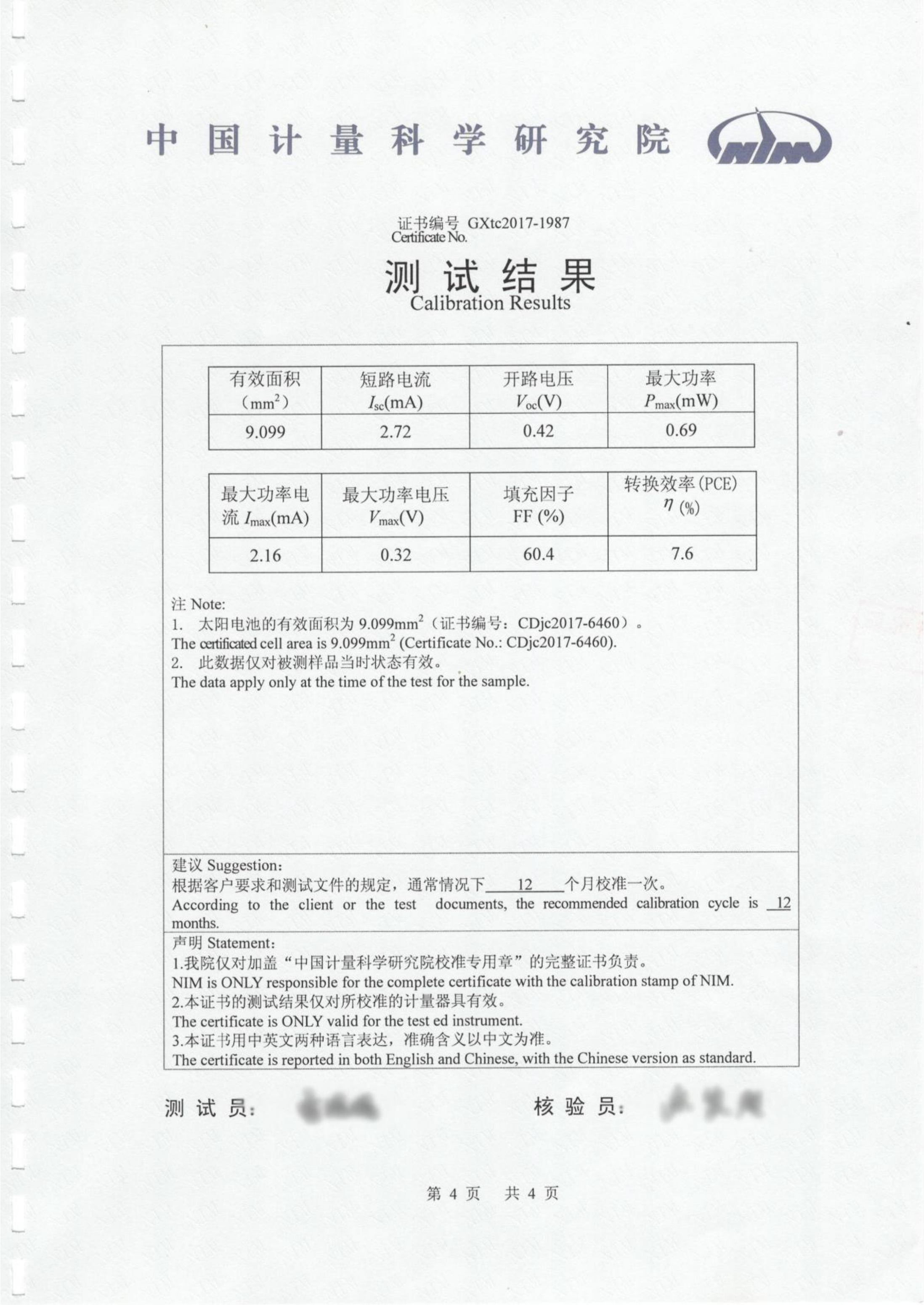

中国计量科学研究院

证书编号 GXtc2017-1987
Certificate No.

测试结果
Calibration Results

有效面积 (mm²)	短路电流 I_{sc}(mA)	开路电压 V_{oc}(V)	最大功率 P_{max}(mW)
9.099	2.72	0.42	0.69

最大功率电流 I_{max}(mA)	最大功率电压 V_{max}(V)	填充因子 FF (%)	转换效率 (PCE) η (%)
2.16	0.32	60.4	7.6

注 Note:
1. 太阳电池的有效面积为 9.099mm²（证书编号：CDjc2017-6460）。
The certificated cell area is 9.099mm² (Certificate No.: CDjc2017-6460).
2. 此数据仅对被测样品当时状态有效。
The data apply only at the time of the test for the sample.

建议 Suggestion:
根据客户要求和测试文件的规定，通常情况下 12 个月校准一次。
According to the client or the test documents, the recommended calibration cycle is 12 months.

声明 Statement:
1.我院仅对加盖"中国计量科学研究院校准专用章"的完整证书负责。
NIM is ONLY responsible for the complete certificate with the calibration stamp of NIM.
2.本证书的测试结果仅对所校准的计量器具有效。
The certificate is ONLY valid for the test ed instrument.
3.本证书用中英文两种语言表达，准确含义以中文为准。
The certificate is reported in both English and Chinese, with the Chinese version as standard.

测试员：[illegible]　　核验员：[illegible]

第 4 页　共 4 页

Details of the device tested and the certificate of our CdS/Sb_2Se_3 device by National Institute of Metrology.

Supplementary Figure 4

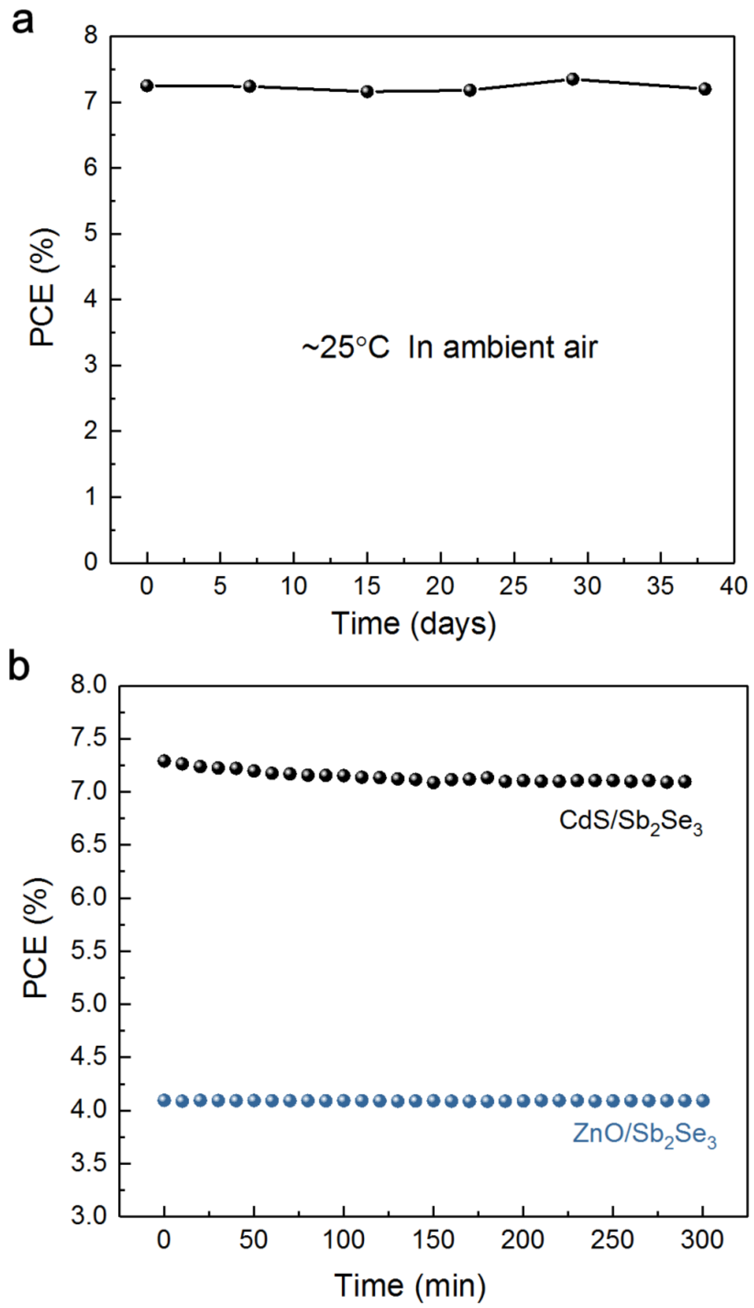

Device stability. (a) the stability of a typical CdS/Sb_2Se_3 device without package stored under regular laboratory conditions (about 25 ℃ in ambient air). (b) Steady-state efficiency of VTD fabricated CdS-based and ZnO-based Sb_2Se_3 devices versus time under continuous 100 mW cm^{-2} AM 1.5G illumination (with maximum power output, in ambient air).

Supplementary Figure 5

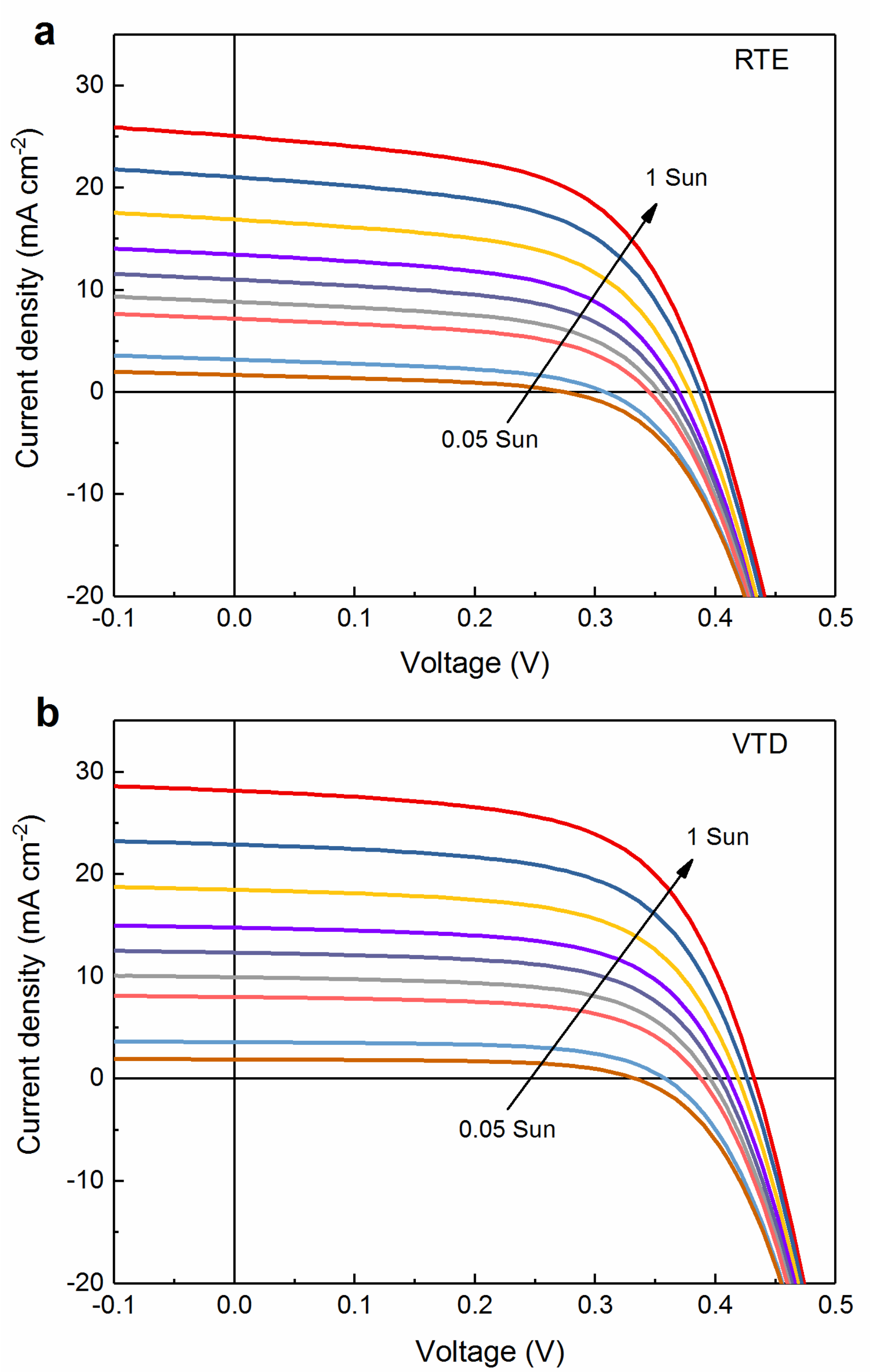

J-*V* curves of (a) RTE and (b) VTD fabricated CdS/Sb_2Se_3 solar cells under different light intensity.

Supplementary Figure 6

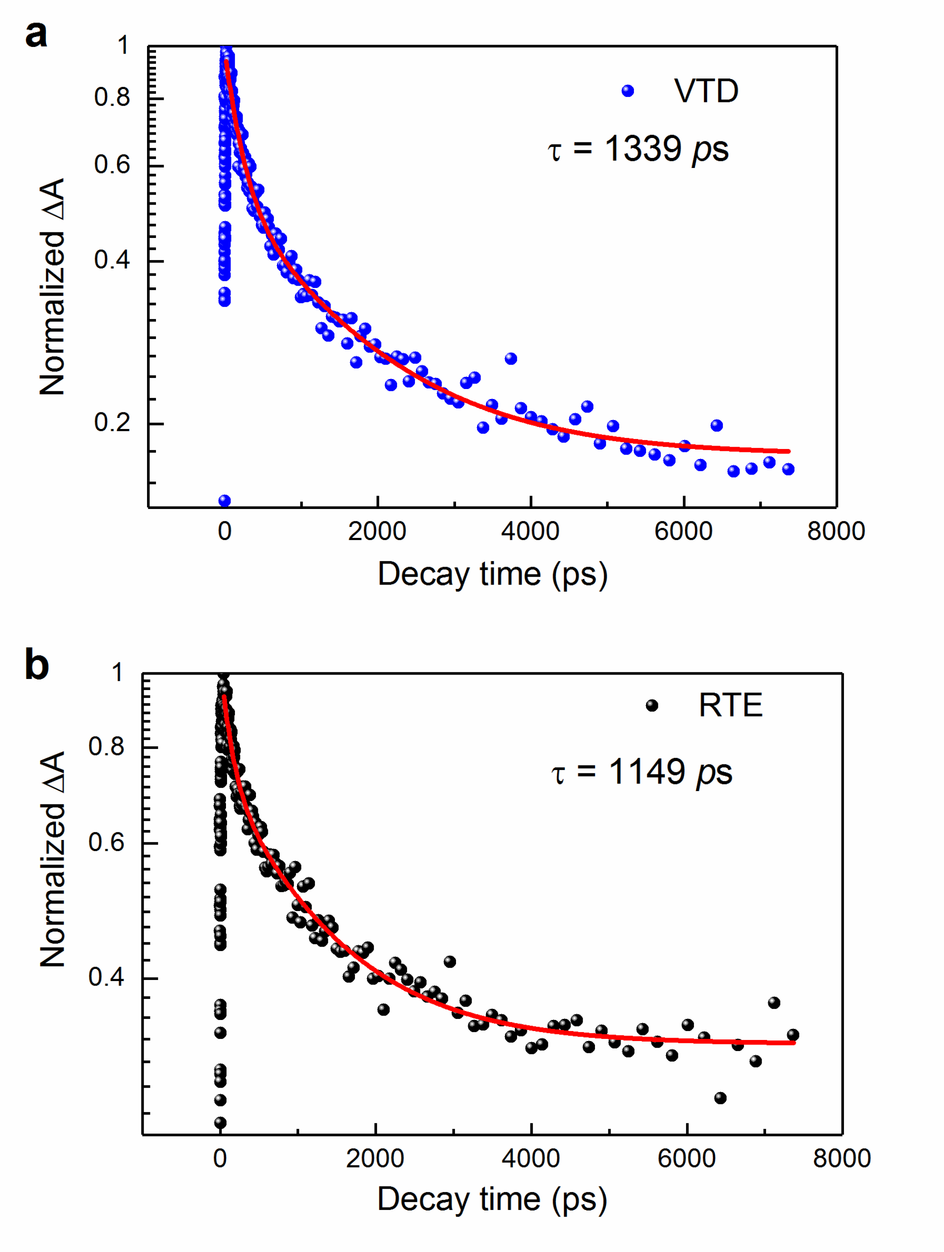

Transient absorption spectroscopy of VTD and RTE fabricated CdS/Sb_2Se_3 solar cells. Transient kinetic decay and kinetic fits (solid lines) monitored at 940 nm for (a) VTD and (b) RTE fabricated devices. Based on the steady-state absorption (Supplementary Fig. 7), the absorption of VTD and RTE fabricated Sb_2Se_3 films rose to maximum at around 940 nm. So we monitored transient kinetic decay at 940nm.

Supplementary Figure 7

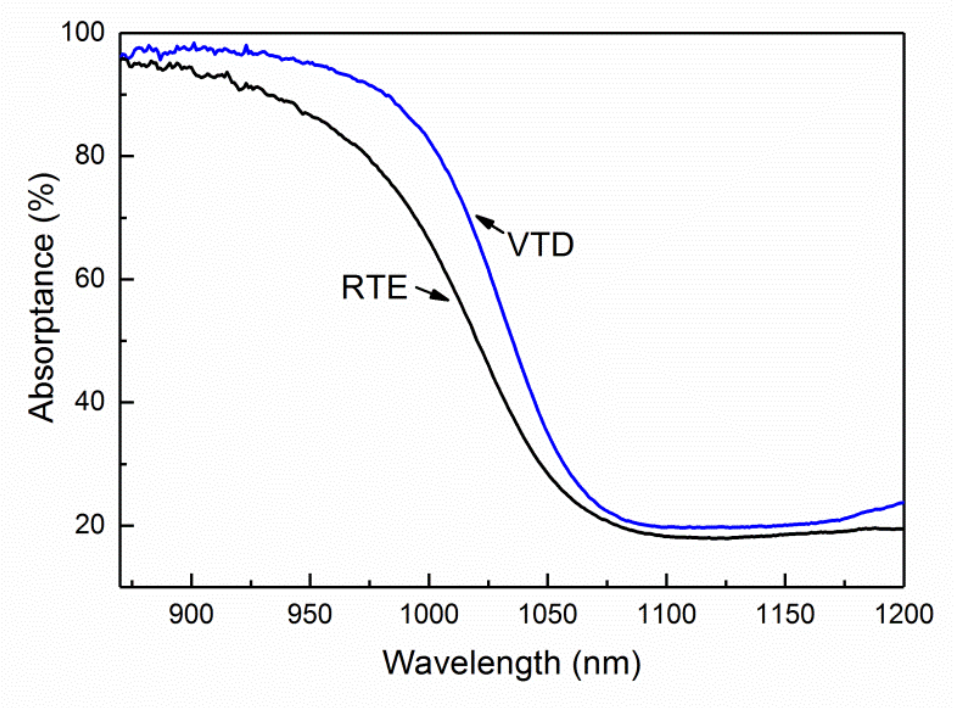

Optical absorptance of VTD and RTE fabricated Sb_2Se_3 films.

Supplementary Figure 8

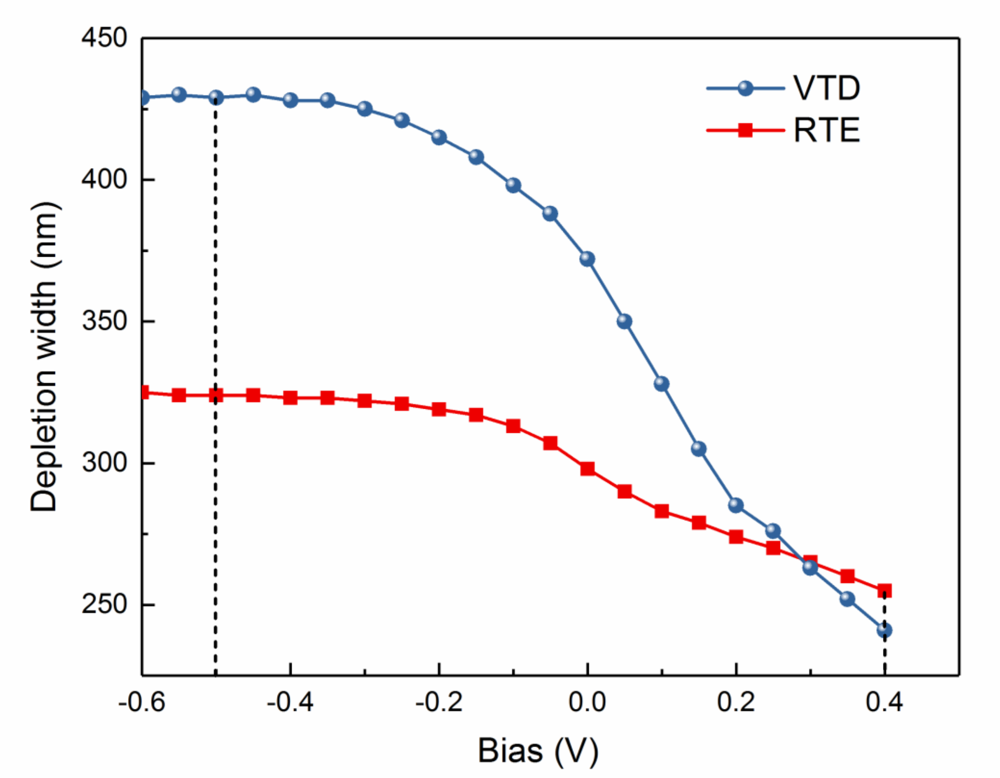

Depletion width *versus* bias curves of VTD and RTE fabricated CdS/Sb_2Se_3 solar cells.

Supplementary Figure 9

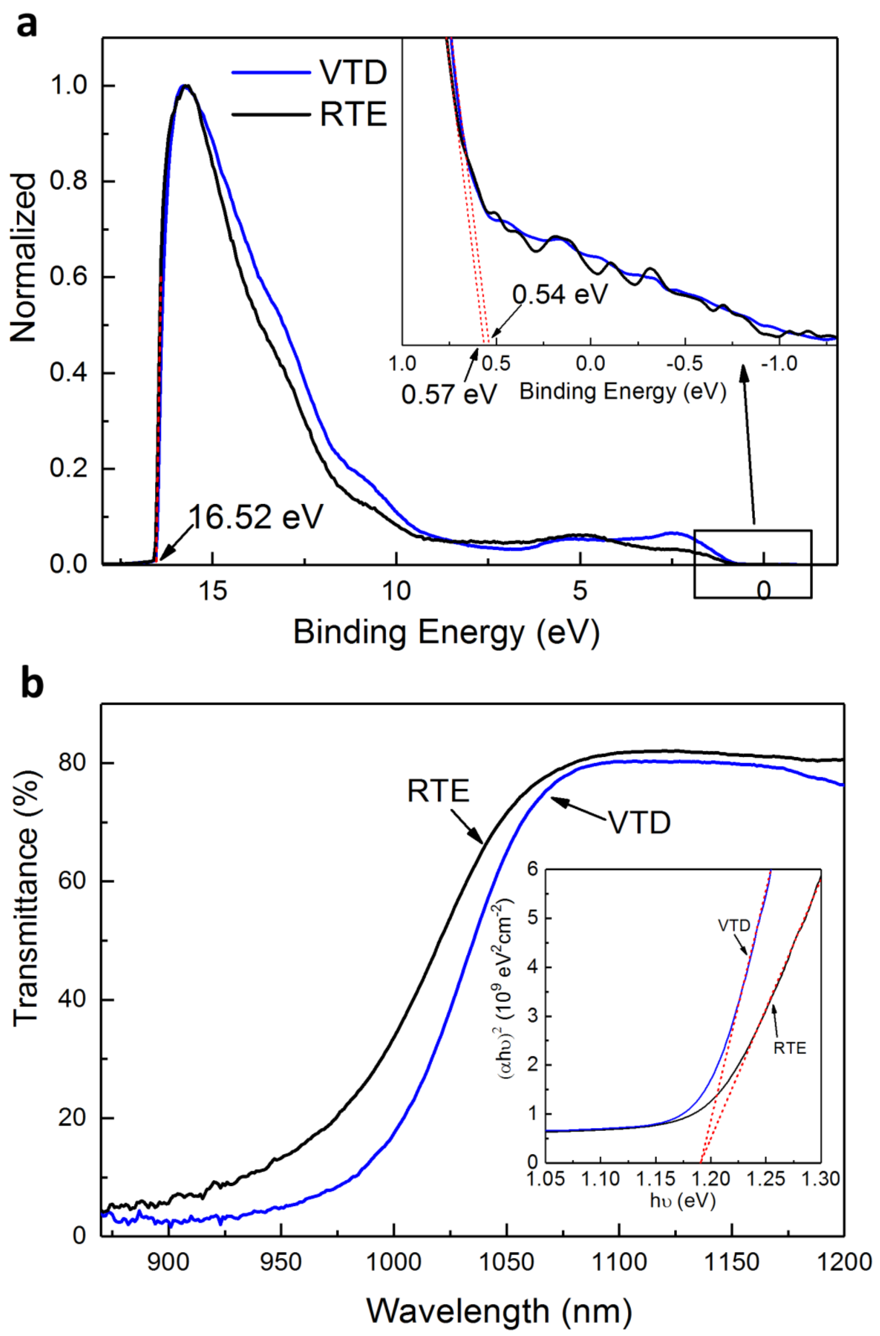

Energy band characterization of VTD and RTE fabricated Sb_2Se_3 films. (a) Ultraviolet photoemission spectroscopy (UPS) spectrum. (b) UV-vis-IR transmittance spectrum and the corresponding Tauc plots for bandgaps.
